# Plasma membrane poration by opioid neuropeptides: a possible mechanism of pathological signal transduction

**DOI:** 10.1038/cddis.2015.39

**Published:** 2015-03-12

**Authors:** O Maximyuk, V Khmyz, C-J Lindskog, V Vukojević, T Ivanova, I Bazov, K F Hauser, G Bakalkin, O Krishtal

**Affiliations:** 1 State Key Lab for Molecular Biology, Bogomoletz Institute of Physiology, Kiev, Ukraine; 2 Department of Clinical Neuroscience Center for Molecular Medicine, Karolinska Institutet, Stockholm, Sweden; 3 Department of Pharmaceutical Biosciences, Uppsala University, Uppsala, Sweden; 4 Department of Pharmacology and Toxicology, Virginia Commonwealth University School of Medicine, Richmond, VA, USA

## Abstract

Neuropeptides induce signal transduction across the plasma membrane by acting through cell-surface receptors. The dynorphins, endogenous ligands for opioid receptors, are an exception; they also produce non-receptor-mediated effects causing pain and neurodegeneration. To understand non-receptor mechanism(s), we examined interactions of dynorphins with plasma membrane. Using fluorescence correlation spectroscopy and patch-clamp electrophysiology, we demonstrate that dynorphins accumulate in the membrane and induce a continuum of transient increases in ionic conductance. This phenomenon is consistent with stochastic formation of giant (~2.7 nm estimated diameter) unstructured non-ion-selective membrane pores. The potency of dynorphins to porate the plasma membrane correlates with their pathogenic effects in cellular and animal models. Membrane poration by dynorphins may represent a mechanism of pathological signal transduction. Persistent neuronal excitation by this mechanism may lead to profound neuropathological alterations, including neurodegeneration and cell death.

Neuropeptides are the largest and most diverse family of neurotransmitters. They are released from axon terminals and dendrites, diffuse to pre- or postsynaptic neuronal structures and activate membrane G-protein-coupled receptors. Prodynorphin (*PDYN*)-derived opioid peptides including dynorphin A (Dyn A), dynorphin B (Dyn B) and big dynorphin (Big Dyn) consisting of Dyn A and Dyn B are endogenous ligands for the *κ*-opioid receptor. Acting through this receptor, dynorphins regulate processing of pain and emotions, memory acquisition and modulate reward induced by addictive substances.^1, 2, 3, 4^ Furthermore, dynorphins may produce robust cellular and behavioral effects that are not mediated through opioid receptors.^5, 6, 7, 8, 9, 10, 11, 12, 13, 14, 15, 16, 17, 18, 19, 20, 21, 22, 23, 24, 25, 26, 27, 28, 29^ As evident from pharmacological, morphological, genetic and human neuropathological studies, these effects are generally pathological, including cell death, neurodegeneration, neurological dysfunctions and chronic pain. Big Dyn is the most active pathogenic peptide, which is about 10- to 100-fold more potent than Dyn A, whereas Dyn B does not produce non-opioid effects.^16, 17, 22, 25^ Big Dyn enhances activity of acid-sensing ion channel-1a (ASIC1a) and potentiates ASIC1a-mediated cell death in nanomolar concentrations^30, 31^ and, when administered intrathecally, induces characteristic nociceptive behavior at femtomolar doses.^17, 22^ Inhibition of endogenous Big Dyn degradation results in pathological pain, whereas prodynorphin (*Pdyn*) knockout mice do not maintain neuropathic pain.^22, 32^ Big Dyn differs from its constituents Dyn A and Dyn B in its unique pattern of non-opioid memory-enhancing, locomotor- and anxiolytic-like effects.^25^

Pathological role of dynorphins is emphasized by the identification of *PDYN* missense mutations that cause profound neurodegeneration in the human brain underlying the SCA23 (spinocerebellar ataxia type 23), a very rare dominantly inherited neurodegenerative disorder.^27, 33^ Most *PDYN* mutations are located in the Big Dyn domain, demonstrating its critical role in neurodegeneration. *PDYN* mutations result in marked elevation in dynorphin levels and increase in its pathogenic non-opioid activity.^27, 34^ Dominant-negative pathogenic effects of dynorphins are not produced through opioid receptors.

ASIC1a, glutamate NMDA (*N*-methyl-d-aspartate) and AMPA (*α*-amino-3-hydroxy-5-methyl-4-isoxazolepropionic acid)/kainate ion channels, and melanocortin and bradykinin B2 receptors have all been implicated as non-opioid dynorphin targets.^5, 6, 7, 8, 9, 10, 11, 12, 13, 14, 15, 16, 17, 18, 19, 20, 21, 22, 23, 24, 25, 26, 30, 31, 35, 36^ Multiplicity of these targets and their association with the cellular membrane suggest that their activation is a secondary event triggered by a primary interaction of dynorphins with the membrane. Dynorphins are among the most basic neuropeptides.^37, 38^ The basic nature is also a general property of anti-microbial peptides (AMPs) and amyloid peptides that act by inducing membrane perturbations, altering membrane curvature and causing pore formation that disrupts membrane-associated processes including ion fluxes across the membrane.^39^ The similarity between dynorphins and these two peptide groups in overall charge and size suggests a similar mode of their interactions with membranes.

In this study, we dissect the interactions of dynorphins with the cell membrane, the primary event in their non-receptor actions. Using fluorescence imaging, correlation spectroscopy and patch-clamp techniques, we demonstrate that dynorphin peptides accumulate in the plasma membrane in live cells and cause a profound transient increase in cell membrane conductance. Membrane poration by endogenous neuropeptides may represent a novel mechanism of signal transduction in the brain. This mechanism may underlie effects of dynorphins under pathological conditions including chronic pain and tissue injury.

## Results

### Dynorphins accumulate in the plasma membrane

**Figure 1:**
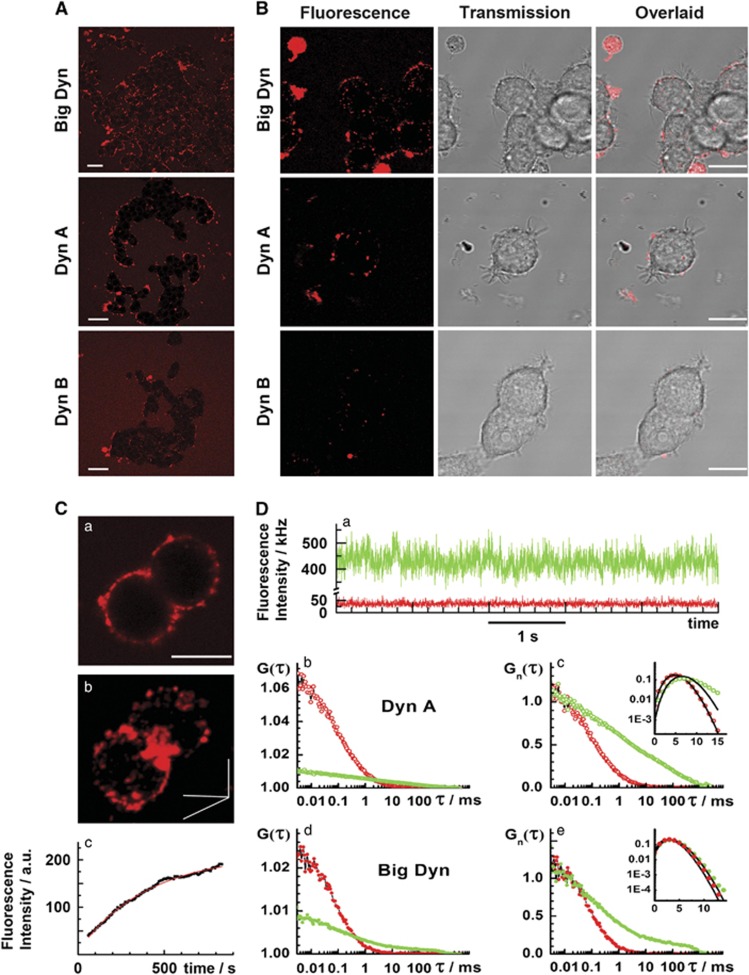
Dynorphins differently accumulate in the plasma membrane of PC12 cells. Confocal fluorescence imaging and FCS show that dynorphin peptides differently interact with live PC12 cells, which do not express opioid receptors.^67^ (**A**) Fluorescently labeled Big Dyn and Dyn A, but not Dyn B, associate with the plasma membrane of live PC12, as evident from the increased intensity of Big Dyn and Dyn A fluorescence in the membrane compared with the medium. Confocal images of live PC12 cells were taken after 30 min incubation with fluorescently labeled dynorphins added to the culture medium at 100 nM concentration. After incubation, the concentrations of TAMRA-Dyn B, TAMRA-Dyn A and TAMRA-Big Dyn in the bulk medium measured by FCS were 100, 70 and 30 nM, respectively. Scale bars, 20 *μ*m. (**B**) Bright fluorescent domains of TAMRA-Big Dyn and TAMRA-Dyn A, but not TAMRA-Dyn B, observed in the plasma membrane of live PC12 cells after dynorphin peptides washout. Scale bars, 10 *μ*m. (**C**, a) Two- (2D) and (b) three-dimensional (3D) distribution of TRITC-labeled Dyn A on the surface of live PC12 cells. Confocal images were taken after 15 min incubation of the cells with 250 nM fluorescently labeled Dyn A. (c) The time course of fluorescence intensity rise at the plasma membrane during 15 min of incubation is shown below (bottom). Scale bar, 10 *μ*m. (**D**) FCS measurements performed after 30 min incubation of PC12 cells with 300 or 150 nM TAMRA-Big Dyn or TAMRA-Dyn A, respectively. (a) Fluorescence intensity fluctuations recorded in the bulk medium (red) and the plasma membrane of live PC12 cells incubated with TAMRA-Dyn A (green). (b) Autocorrelation curves reflecting TAMRA-Dyn A dynamics and concentration in the bulk medium (red dots) and the plasma membrane of live PC12 cells (green dots) showing that TAMRA-Dyn A accumulates in the plasma membrane, as evident from the increased average number of molecules in the plasma membrane, *N*_pm_=(105±15), as compared with the medium, *N*_med_=(20±2). (c) Autocorrelation curves recorded in the medium (red) and the plasma membrane (green) normalized to the same amplitude (*G*_n_(*τ*)=1 at *τ*=10 *μ*s), showing a marked shift of the autocorrelation curve towards longer characteristic times because of TAMRA-Dyn A interactions with, and its reduced mobility in, the plasma membrane. *Inset:* PCHs show that photon counting distribution recorded in the plasma membrane (green circles) deviates from the Poisson distribution (black line, mean=5.17) more than the photon counting distribution observed in the cell culture medium (red circles). (d) Autocorrelation curves reflecting TAMRA-Big Dyn dynamics and concentration in the bulk medium (red dots) and the plasma membrane of live PC12 cells (green dots). The average number of TAMRA-Big Dyn molecules at the plasma membrane, *N*_pm_=(119±19), is several times higher than the average number of molecules in the bulk cell culturing medium, *N*_med_=(45±6). (e) Autocorrelation curves normalized to the same amplitude (*G*_n_(*τ*)=1 at *τ*=10 *μ*s) show that TAMRA-Big Dyn lateral mobility is significantly reduced, as evident from the appearance of a second component with a significantly longer diffusion time. *Inset:* PCHs show that photon counting distribution recorded in the plasma membrane (green dots, mean=3.93) deviates more from the Poisson distribution (black line, mean=3.68) than the photon counting histogram recorded in the cell culture medium (red dots), which could be fitted by Poisson distribution (black line, mean=3.16)

Confocal laser scanning microscopy (CLSM) imaging with single-molecule sensitivity^40^ allows visualization of fluorescently labeled dynorphins in the cells and surrounding medium (Figure 1A). Fluorescence imaging showed that TAMRA-Big Dyn and TAMRA-Dyn A, but not TAMRA-Dyn B, accumulated in the plasma membrane after 30 min incubation, forming a punctate bright fluorescent rim at the cell/medium interface (Figures 1A–C). All three peptides were also seen in the medium surrounding the cells, appearing as background in the images shown in Figure 1A. Initial concentrations of all three peptides were 100 nM, but after 30 min incubation, TAMRA-Big Dyn and TAMRA-Dyn A concentrations in the medium were substantially decreased from the initial 100 nM to 30 and 70 nM, respectively, whereas that of [TAMRA-Dyn B] remained virtually unchanged (compare Figure 1A with Supplementary Figure S1). Preferential localization in the membrane was evident over a wide range, from 10 to 500 nM of [TAMRA-Big Dyn] and [TAMRA-Dyn A] in the medium, and for different incubation times, from 15 min to several hours. Peptide distribution on the membrane was not uniform, but characterized by the formation of bright domains (Figures 1B and C). The domains were seen in cells incubated with fluorescently labeled peptides for 15 min (Figure 1C) and could be observed for at least 2 h after washing out fluorescent dynorphins and incubating in a peptide-free cell culture medium (Figure 1B). Figure 1C shows confocal image of PC12 cells with the two- and three-dimensional patchy distribution of fluorescent Dyn A in the plasma membrane. The bright Dyn A domains were limited in size by the diffraction of light (~200 nm), suggesting that their actual sizes may be smaller. In contrast, cell debris was homogeneously stained (see large bright spots in Figure 1A).

The local concentration of TAMRA-Dyn A and TAMRA-Big Dyn in the plasma membrane exceeded several times their concentration in the bulk cell culture medium, as evident from the fluorescence intensity fluctuations recorded in the plasma membrane and the medium (Figure 1Da) and from the two- to sixfold difference between the amplitudes of autocorrelation curves recorded in the medium and on the cell surface (Figures 1Db and d). Mobility of TAMRA-Dyn A and TAMRA-Big Dyn at the cell surface was significantly reduced compared with their mobility in the cell culture medium, as shown by the shift of the autocorrelation curves towards longer characteristic times (Figures 1Dc and e).

Photon counting histograms (PCHs) (insets in Figures 1Dc and e) showed a shift in the distribution of photon counts to higher values for measurements at the plasma membrane (green) as compared with the measurements in the cell culture medium (red). In addition, a broadening of the PCH and larger deviation from Poison distribution (represented by the black line in Figures 1Dc and e) were also observed. This is consistent both with an increase in the average number of molecules and an increase in molecular brightness. The contribution of these processes could not be discerned because of the absence of reliable standards. Thus, fluorescence imaging and FCS demonstrate that fluorescently labeled Big Dyn and Dyn A, but not Dyn B, associate with the plasma membrane, forming distinct bright domains at the cell surface.

### Dynorphins produce profound stochastic surges in membrane conductance

**Figure 2:**
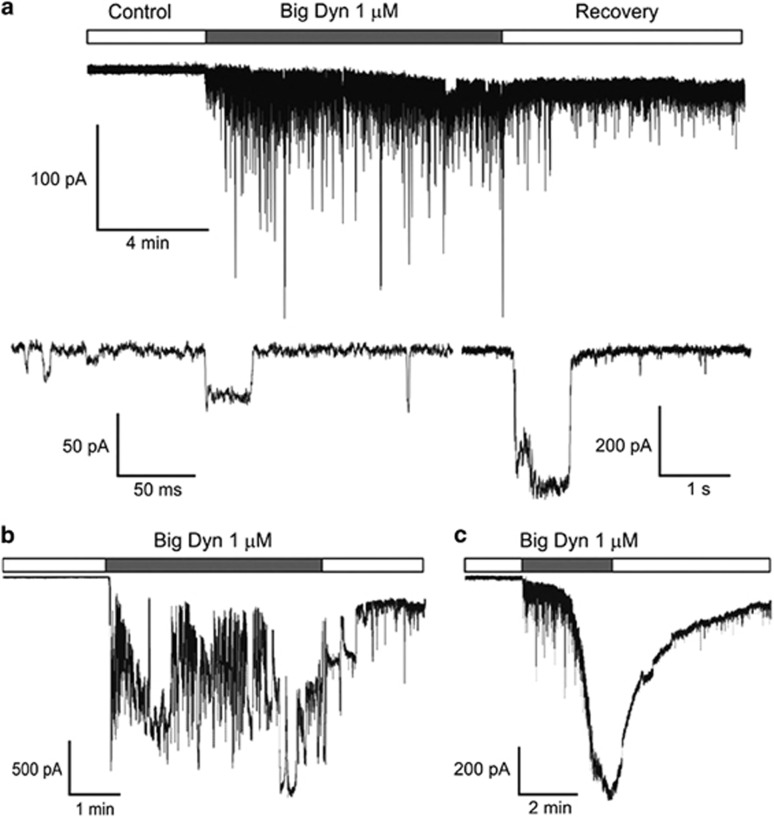
Big Dyn induces fluctuations in the plasma membrane conductance of DRG neurons. (**a**, top) Representative transmembrane holding current induced by 1 *μ*M Big Dyn observed in ~85% of tested neurons. (**a**, bottom) Application of Big Dyn resulted in the appearance of current surges strongly varying in duration and amplitude (note the different time scales). (**b** and **c**) Alternative dynamical behavior observed in about ~15% of cells. All measurements were conducted on DRG neurons clamped at −100 mV

The effects of dynorphins on plasma membrane were examined using standard patch-clamp technique. Extracellular application of 1 *μ*M Big Dyn at holding voltage −100 mV induced profound increase in membrane noise visible as current fluctuations (Figure 2a) strongly varying both in amplitude (from 20 pA to 1.7 nA) and duration (from 2 ms to 1.5 s). These current surges usually developed after a delay that decreased with increasing concentrations of Big Dyn, but this differed greatly among cells. The observed effect was slightly diminished upon Big Dyn removal but full recovery was not observed even after continuous perfusion of the cell with Big Dyn-free solution for 1 h. In most cases, ~85% (*n*=87), cells responded to the application of Big Dyn as shown in Figure 2a, but ‘non-typical' effects were occasionally observed (stochastic or smooth changes in the current base line, Figure 2b, ~10%, *n*=9 and Figure 2c, ~5%, *n*=5), resulting in catastrophic cell membrane breakdown; such cells were not considered in subsequent analyses. Big Dyn produced similar effects on various neurons (dorsal root ganglia (DRG, *n*=101), hippocampal pyramidal neurons (*n*=8), cerebellum Purkinje cells (*n*=9)) and on non-excitable human embryonic kidney 293 (HEK293) cells (*n*=34).

**Figure 3:**
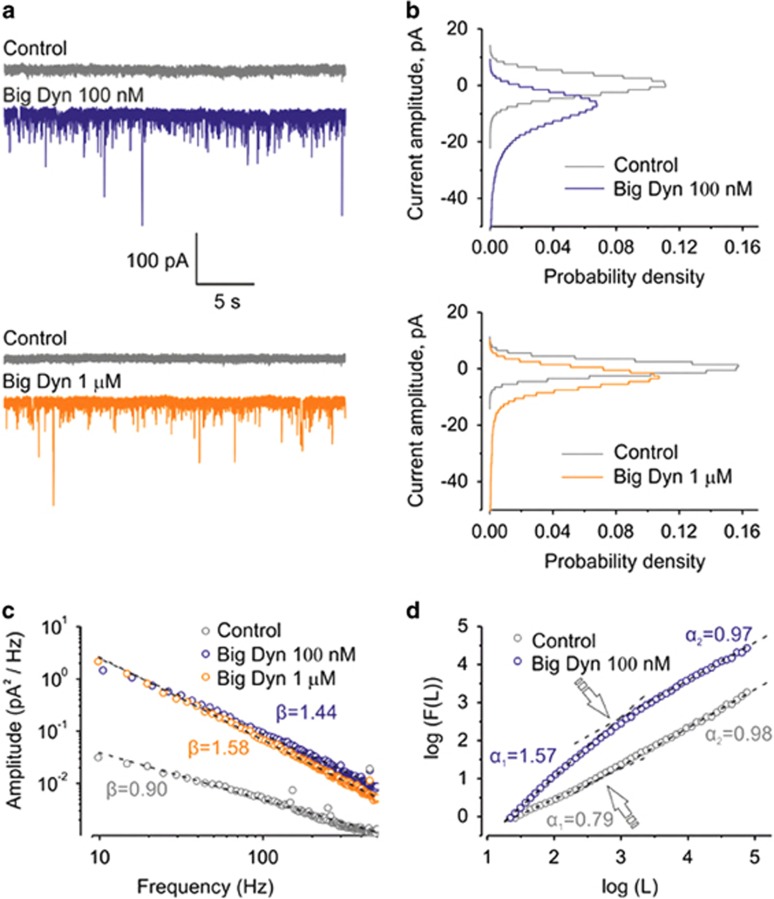
Properties of Big Dyn-induced current surges in DRG neurons. (**a**) Current surges across the plasma membrane recorded in control cells (gray) and induced by incubation with 100 nM Big Dyn (blue trace) or 1 *μ*M of Big Dyn (orange race). (**b**) All-point histograms for the holding currents as shown in (**a**). (**c**) Power-law decay measured in the power spectrum (*S*(*f*)~1/*f^β^*) of basal transmembrane current noise. Under control conditions, the power-law decaying power spectra close to 1/f was observed (gray trace). In response to Big Dyn, the power spectrum tended towards Brownian noise spectra, characterized by larger decay exponents *β* (orange and blue traces). (**d**) Self-affinity of a signal calculated by DFA expressed as log–log plot *F*(*L*) *vs* *L*, shows crossovers indicated by arrows in both control conditions and under Big Dyn. All measurements were conducted on DRG neurons clamped at −100 mV

Big Dyn-induced membrane current surges (Figure 3a, blue and orange traces) seemingly resembled single channel-like activity. However, discrete current levels characteristic of single-channel behavior were not observed in the corresponding all-point amplitude histograms (Figure 3b). Similar single-peak all-point histograms were obtained for both 100 nM and 1 *μ*M Big Dyn concentrations (Figure 3b, blue and orange histograms), but onset time of the effect was substantially longer for the lower concentration (Supplementary Figure S2). Because time-domain analysis did not reveal any sign of single channel-like activity in any of the cells tested (*n*=23), we analyzed Big Dyn-induced signal in the frequency domain. Figure 3c shows power-law decaying power spectrum (*S*(*f*)~1/*f^β^*) calculated from the basal transmembrane current noise in comparison with the spectra under Big Dyn. In the absence of Big Dyn (Figure 3c, gray curve), the spectral exponent was *β*=0.90, which is close to its value for the pink noise (1/*f*, *β*=1) that is characteristic of many biological and physical systems. In the case of neuronal membrane, the holding current comprises contributions of numerous ionic conductances. Their random fluctuations occur at different points and on different time scales, yielding basal membrane 1/*f* noise.^41, 42, 43^ Under Big Dyn treatment, the noise power gradually increased (Figure 3c, blue and orange curves) and the spectral exponent attained higher values, *β*=1.44 for 100 nM and *β*=1.58 for 1 *μ*M. Similar to the previous case, the onset of the effect was substantially slower for the lower concentration (Supplementary Figure S3). Thus, the treatment with Big Dyn resulted in the appearance of non-stationary power-scaled noise signal, which could not be because of single-channel-like activity and therefore could not be analyzed by conventional methods.

To analyze such complex power-scaled signals, we used detrended fluctuation analysis (DFA, see Materials and Methods), a recently introduced approach for analyzing non-stationary signals.^44^ DFA determines the statistical self-affinity of a signal, which is described by the parameter *α* (see Materials and Methods for detailed description). The value of *α*=1/2 corresponds to uncorrelated white noise, whereas the value of *α* exceeding 1/2 indicates the existence of positive correlations in the signal; *α*≈1/2 corresponds to pink noise and 3/2 to Brownian noise. Thus, DFA quantifies fractal-like autocorrelation properties of signals allowing to determine which signal is more regular and less complex, that is, to discriminate between ‘healthy' and ‘pathological' data.^45^ Figure 3d shows log–log plot of fluctuations *F*(*L*) from a single cell, under control conditions and in the presence of 100 nM Big Dyn *versus* the time window *L* for time series shown in Figure 3a. Consistent with previous studies on biological subjects, a crossover phenomenon was always observed in the log–log plot *F*(*L*) *vs L* both under control condition and in the peptide presence. This prompted us to extract two parameters from each data set by fitting the scaling exponent *α* over two different, ‘short' and ‘long', time scales, similarly as it was done by Peng *et al.*^44^ for the analysis of heart dynamics. For each data set, we calculated an exponent *α*_1_ by making least squares fit of log(*F*(*L*)) *vs* log(*L*) for the ‘milliseconds time scale' (2–30 ms). Similarly, an exponent *α*_2_ was obtained for the ‘seconds time scale' (0.5–7.5 s). In summary, we obtained two statistically different scaling exponents *α*_1_=0.79±0.09 and *α*_2_=0.98±0.06 (*n*=18; *P*<0.0001) under control conditions. Although slightly different, both exponents are close to 1, indicating similar statistical self-affinity of the signal at both time scales. However, in the presence of Big Dyn, *α*_1_ increased to 1.25±0.17 (*n*=15; *P*<0.0001), whereas *α*_2_ remained unchanged (1.0±0.08, *n*=15; *P*=0.9998). Thus, following exposure to Big Dyn, membrane conductance noise significantly changes its properties demonstrating transition from 1/*f* fluctuations to Brownian noise consistent with random walk-like process that occurs at a millisecond time scale. In summary, DFA qualitatively confirms the results of spectral analysis, as expected from the Wiener–Khinchin theorem.^46^

**Figure 4:**
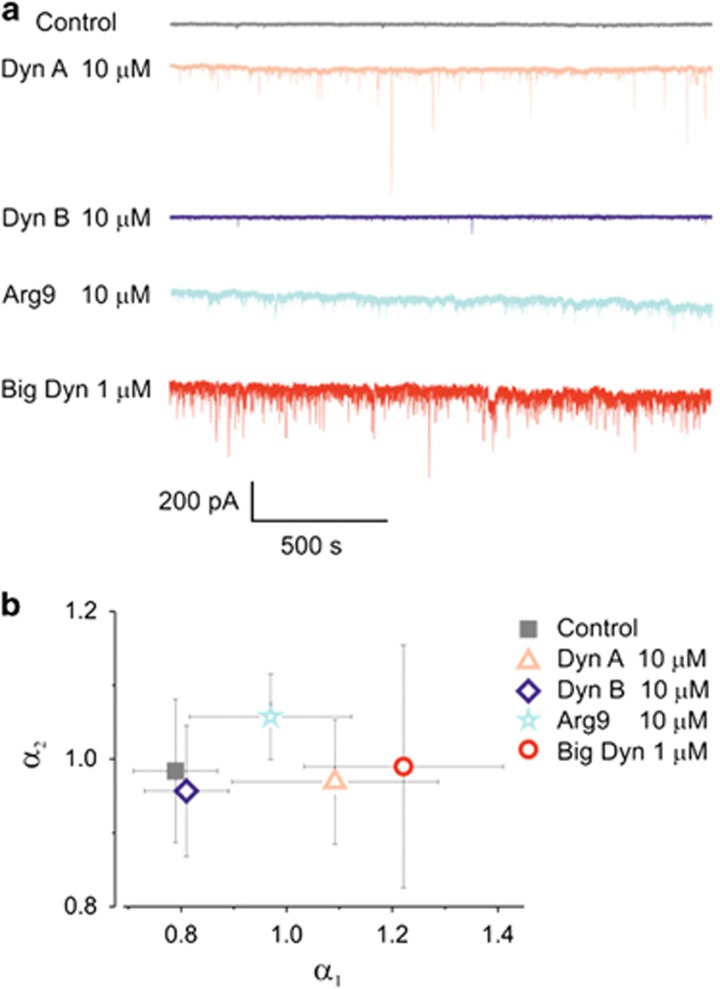
Activity of dynorphins is compared with the activity of synthetic nona-arginine (Arg9). (**a**) Representative recordings of membrane current noise measured in DRG neurons. (**b**) Summary results for scaling exponents *α*_1_ *vs α*_2_ under different peptides show their activity profile. All data were obtained from DRG neurons clamped at −100 mV

Recent studies have demonstrated that arginine-rich peptides generate negative Gaussian membrane curvature with the potency directly related to their arginine content.^47, 48^ We compared the potency of Dyn A, Dyn B, Big Dyn and nona-arginine (Arg9) to induce membrane current fluctuations (Figure 4). Application of 10 *μ*M Dyn A (three arginines) induced clearly visible membrane current fluctuations and corresponding increases in the scaling exponent *α*_1_ from 0.79±0.09 to 1.14±0.22 (*n*=4; *P*<0.001) (Figure 4a), whereas equimolar concentration of Dyn B, which only contains two arginine residues, did not induce detectable changes in the current events and insignificantly affected self-similarity of membrane noise (*α*_1_=0.81±0.08; *n*=4; *P*=0.9997). Big Dyn (six arginines) induced most profound changes in the cellular membrane conductance as well as largest increase in the scaling exponent *α*_1_ (*α*_1_=1.25±0.17; *n*=15; *P*<0.0001), as summarized in Figure 4b. It should be noted that, contrary to all tested dynorphins, Arg9 affected both scaling exponents, *α*_1_ and *α*_2_, which may reflect a different mechanism of cell membrane perturbation as compared with dynorphins.

Big Dyn-induced current noise was not affected by coapplication of the general opioid antagonist naloxone (100 *μ*M), whereas N-terminally truncated Dyn A (des-Tyr-Dyn A), which does not activate opioid receptors, produced effects similar to those of Dyn A, albeit at higher concentrations (Supplementary Figure S4). This clearly indicates that the observed phenomena are not mediated *via* opioid receptors. Notably, the composition of the extracellular solution (see subsection Electrophysiology in Materials and Methods) virtually excluded intracellular metabotropic processes including G-protein signaling, thus ruling out the involvement of opioid or bradykinin receptors.^24^

### Size of dynorphin-induced pores

**Figure 5:**
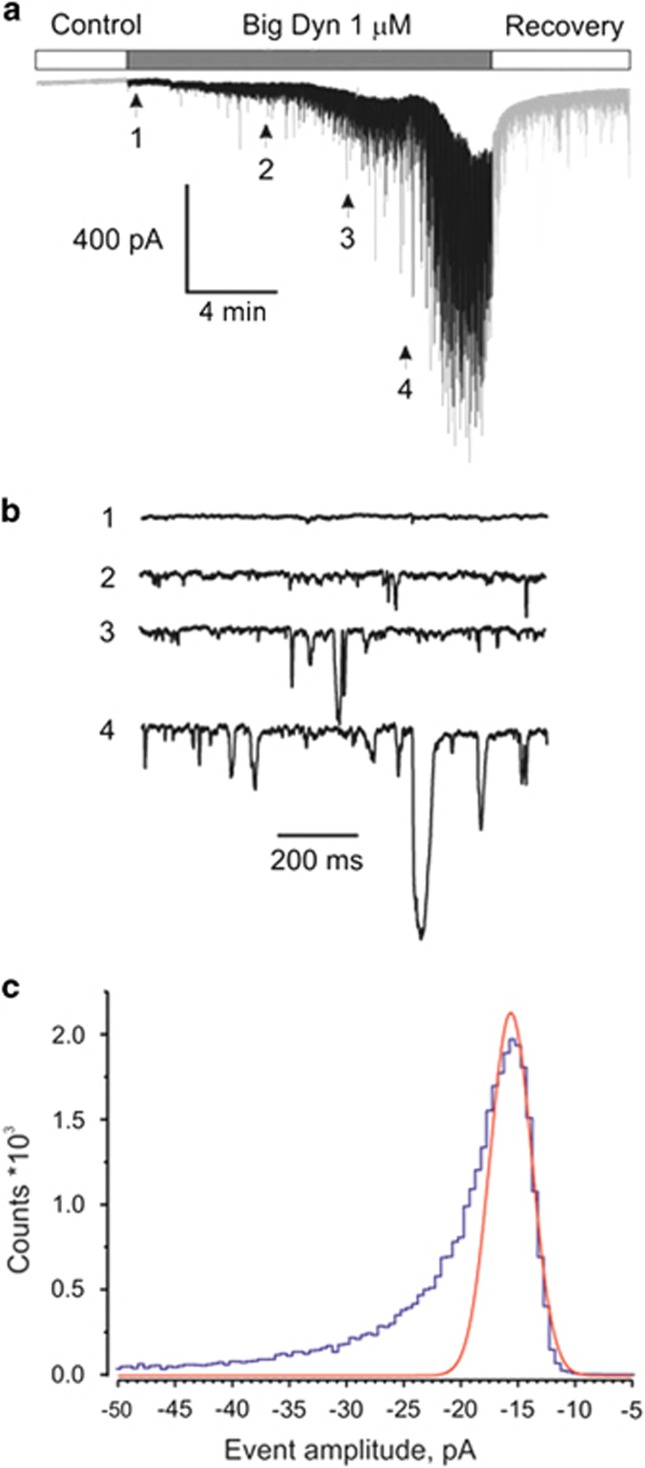
Pore size estimation. (**a**) Application of 1 *μ*M Big Dyn at holding voltage of −100 mV elicited the appearance of progressively increasing basal current noise of HEK293 cells. (**b**) Indicated fragments of basal current recording from (**a**) are shown in milliseconds time scale. (**c**) All current surges under Big Dyn from the cell shown on (**a**) were used for estimation of event mode amplitude, resulting mode diameter of pore near 2.7 nm. The holding voltage was −100 mV, NMDG-HEPES solutions (see Materials and Methods) was used in this experiment to minimize errors connected with activities of basal ionic conductances

Substitution of small ions for the large ones (HEPES^−^ and NMDG^+^, NMGD-HEPES solution) does not prevent the appearance of Big Dyn-induced events pointing at the absence of ion specificity of the observed conductance changes (Figures 5a and b). Assuming that these events are associated with pore formation in the membrane, we have roughly estimated the most probable diameter of the pores by measuring mode conductance value (Figure 5). Assuming that the pore is a uniform cylinder of radius *r*, height *h* and that intra- and extracellular solutions have resistivity *ρ*, we can express pore resistance as *R*_pore_ = (*h*+(*ρr*)/2)*ρ*/*πr*^2^ using the model proposed by Hille.^49^ As the pore conductance *g* is the inverse of *R*_pore_ and solution conductivity *σ* is the inverse of resistivity *ρ*, the above equation for *R*_pore_ can be rearranged to determine pore diameter *d*, from: 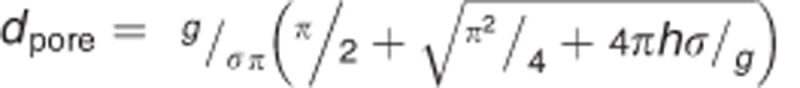 The mode pore conductance *g* (16 pA/100 mV=0.16 nS) was estimated from the amplitude histogram (Figure 5c), the conductivity of NMDG-HEPES solutions was ~0.26 S/m and pore height *h* was estimated as the typical cell plasma membrane thickness of ~7 nm. These estimates resulted in the mode pore diameter of ~2.7 nm.

## Discussion

This study demonstrates that dynorphin neuropeptides Big Dyn and Dyn A efficiently accumulate in the plasma membrane of several types of mammalian cells and induce powerful changes in membrane conductance, which is consistent with the appearance of large pores that produce nonspecific ion exchange. These effects are not mediated by opioid receptors, which is evident from the experiments with naloxone and des-Tyr-Dyn A (Supplementary Figure S4) as well as from the experiments with HEK293 cells that do not express opioid receptors. The involvement of metabotropic receptors in the observed phenomena may also be ruled out, because they are not functional in the extracellular solutions used in the experiments.

Bright dynorphin complexes on the plasma membrane identified by fluorescence imaging and FCS may represent the membrane ion-conducting pores demonstrated by patch clamp. Although direct evidence is missing, several observations support this notion. Consistent with FCS data, correlations in ms time scale were found in the dynorphin-induced signal. Molecular complexes of dynorphins enriching the plasma membrane may form quasistable pores in it similar to polyarginines^50^ or generate negative Gaussian membrane curvature with the efficiency directly related to their arginine content.^47, 48^ Both mechanisms result in the stochastic appearance of large nonselective ion conductances detectable as holding current surges of various amplitudes and durations. The oligomerization of amyloid peptides and AMPs have been demonstrated to trigger membrane leakage.^39, 51, 52^ By analogy, oligomeric dynorphin assemblies identified by the fluorescence imaging and FCS may be responsible for membrane perturbation and pore formation.

Using DFA, we have found that Big Dyn induces non-stationary stochastic noise, which has power-law decaying spectral density *S*(*f*)~1/*f^β^* and asymmetric heavy-tailed single-peak probability density function (all-points histogram) that is indicative of the absence of discreet current levels. These data suggest that dynorphins promote the stochastic appearance of unstructured pores in the cellular membrane that have no similarity with typical high-order macromolecular membrane assemblies such as ion channels, transporters, and so on.

The estimated Big Dyn-induced pore (2.7 nm) is compatible with the diameter of malaria parasite's nutrient channel (2.3 nm),^53^ and the diameter of recombinant large-conductance mechanosensitive ion channel (4.0 nm) from *Escherichia coli*; the latter is the largest ion channel described so far.^54^ Not surprisingly, these pores are capable of conducting large organic ions such as HEPES^−^ and NMDG^+^ (approx. molecular radius 0.4 nm). Such observations are in a good agreement with the model experiments demonstrating that dynorphin induces leakage of calcein molecules with 0.65 nm radius from unilamellar phospholipid vesicles.^55^

Among compounds capable to induce membrane perturbations and/or formation of transmembrane pores, dynorphins are similar to AMPs, amyloid peptides (including islet amyloid polypeptide), venom peptide toxins and cell-penetrating peptides (CPPs).^39, 56, 57^ All these peptides are basic, which seems to be critical for their membrane effects. Indeed, the membrane actions of dynorphins correlate with the arginine content (Table 1 and Figure 4). Interactions of AMPs and CPPs with the membrane also depend on a combination of physicochemical and structural features, such as size, residue composition, secondary structure, hydrophobicity and amphiphilic character.^56^ The peptide size and the presence of aromatic or hydrophobic residues are critical too. Thus, effects of both long Big Dyn and Dyn A were much stronger as compared with the effects of a short, very basic model Arg9 peptide, and the deletion of the N-terminal Tyr resulted in marked decrease of Dyn A pore-making potency.

The extent to which dynorphins accumulate in the plasma membrane correlate with their ability to alter membrane conductivity; Big Dyn demonstrates greater accumulation and potency than Dyn A, whereas Dyn B is largely inactive. Importantly, this rank order coincides with the dynorphin ability (i) to generate pores in model phospholipid vesicles,^55, 58, 59^ (ii) to translocate across the cell membrane^55, 58, 59^ and (iii) to induce behavioral effects including pathological pain.^17, 22, 25^ Altogether, these results strongly infer a causal connection between the dynorphin-induced pore formation in the plasma membrane and the *in vivo* dynorphin actions.

In the human hippocampus, dynorphins are mostly accumulated in the mossy fibers and to some extent in granule cell dendrites.^2^ During epileptic seizures, extensive release of dynorphins apparently occurs, and, as a feedback, the released dynorphins may inhibit seizures through the activation of *κ*-opioid receptors.^60, 61^ Paradoxically, there is mismatch in localization of dynorphins and *κ*-opioid receptor that is expressed in medial CA1 and subiculum.^62^ At the same time, the dynorphin localization overlaps with the areas demonstrating the seizure-induced cell loss.^2, 62^ These observations and our findings suggest that Big Dyn and Dyn A, which concentrations are high at the sites of their release may damage cells by acting through noncanonical, non-opioid mechanism.

Our data indicate that dynorphins accumulate in the neuronal membrane and form large nonselective transient pores as revealed by electrophysiological measurements. This may also trigger the activation of second messenger systems, voltage-dependent Ca^2+^ and Na^+^ channels, post-translational modification of ion channel or receptor proteins and neuronal activation. Besides ions, these pores may pass large molecules such as ATP or even RNAs and proteins enabling exchange of metabolites and regulatory molecules between the axo- and dendroplasm and the extracellular milieu.

We hypothesize that the membrane dynorphin actions represent a novel mechanism of pathological signal transduction. Persistent activation of this mechanism may contribute to pathological processes in the nervous system. Further insights into inhibiting poration caused by dynorphins may have implications for design of novel therapeutics that can limit pathogenic effects of dynorphins.

## Materials and Methods

### Cell culture

PC12 cells were cultivated under standard cell culture conditions, at 37 °C in humidified air with 5% CO_2_, in RPMI 1640 medium (Gibco, Carlsbad, CA, USA; no. 21875-034) supplemented with 10% horse serum (Gibco; no. 26050-088), 5% fetal bovine serum (FBS; Gibco; no.10100-147) and 1% penicillin/streptomycin (Gibco; no. 15140-122). For fluorescence imaging and FCS, the cells were transferred from the culturing flasks to 8-well chambered cover glass (NalgeNunc International, Rochester, NY, USA; no. 155409) one day before observation and cultured in a phenol red-free medium (RPMI 1640; Gibco; no. 11835-063) completed in the same way as described above. PC12 cell confluence in individual wells was about 50–70%.

Primary cultures of DRG neurons were prepared from 8- to 12-day-old Wistar rats of both sexes.^63, 64^ After decapitation, ganglia were rapidly removed and transferred into minimum essential medium Eagle's solution (MEM) containing 4 mg/ml trypsin and 2 mg/ml collagenase and incubated for 25 min at 35 °C. Throughout the entire procedure, the medium was continuously saturated with a 95% O_2_/5% CO_2_ gas mixture to maintain pH at 7.4. After the enzyme treatment, ganglia were washed out and dissociated in the MEM solution containing 10 mM of HEPES-NaOH (pH 7.4). Suspension of isolated neurons was transferred into 25 mm Petri dishes containing: 90% Dulbecco's modified Eagle's medium (DMEM), 0.3% penicillin, 10 *μ*g/ml insulin and 10% fetal calf serum (pH=7.4; when incubated in 95% air/5% CO_2_). After maintaining for 5–48 h at 37 °C, the cells were used in electrophysiological experiments.

HEK293 cells (American Type Culture Collection, Manassas, VA, USA) were cultured in DMEM supplemented with 10% FBS and 10 U/ml penicillin and 10 mg/ml streptomycin (all from Invitrogen, Carlsbad, CA, USA). Dissociated cells were either replated for a new passage or used for patch-clamp experiments. Cells were cultured at 37 °C under an atmosphere of 5% CO_2_ and 95% air with 95% humidity.

### Confocal imaging and fluorescence correlation spectroscopy

CLSM images were obtained by the LSM 510 ConfoCor 3 system (Carl Zeiss, Jena, Germany) individually modified to enable the use of avalanche photodiodes (SPCM-AQR-1X; Perkin-Elmer, Waltham, MA, USA) for imaging. These detectors are characterized by higher sensitivity and lower noise levels, which makes it possible to visualize fluorescently labeled dynorphin molecules at nanomolar concentrations.^40^ TAMRA or TRITC were used as fluorescent markers. No principal differences between the effects of TAMRA- or TRITC-labeled dynorphins were observed. TAMRA or TRITC were excited using the HeNe 543 nm laser. The HFT 488/543/633 main dichroic beam splitter was used to separate the incident and emitted light. Emitted fluorescence was transmitted to the detector through a long-pass filter LP580. The pinhole size in front of the detector was 80 *μ*m (1 Airy). Images were acquired using the C-Apochromat × 40, NA=1.2, water immersion UV-VIS-IR objective, scanning speed 25.6 *μ*s/pixel, without averaging and 512 × 512 pixel resolution.

Fluorescence correlation spectroscopy (FCS) measurements were performed using the same optical pathway as for imaging (described above). Fluorescence intensity fluctuations were recorded in a series of 10 consecutive measurements, each measurement lasting 10 s. Temporal autocorrelation analysis was used to analyze the fluorescence intensity fluctuations to determine the concentration and the diffusion time of the investigated species in water, cell culturing medium, plasma membrane, cell nucleus and the cytoplasm. The online program VassarStats (http://vassarstats.net/poissonfit.html) was used for PCH fitting. FCS background and additional information on data analysis is provided in the Supplementary Figures S5 and S6.

Confocal imaging and FCS experiments were performed at both room temperature (21 °C) and ambient atmosphere (air), or under controlled temperature (37 °C) and atmosphere (5% CO_2_ in humidified air). In the latter case, temperature and atmosphere in cell culture were continuously monitored and regulated at the microscope using a heated microscope stage (Heating insert P, PeCon GmbH, Erbach, Germany), incubator box (Incubator S, PeCon GmbH), atmosphere controlling device (CTI-Controller 3700, PeCon GmbH) and a temperature-controlling device (Tempcontrol 37–2 digital, PeCon GmbH). The data obtained at these conditions were essentially the same; those performed under controlled ambient and atmosphere conditions are shown.

Fluorescently labeled opioid peptides were custom synthesized and purified >98% by Biomatik (Wilmington, DE, USA). To obstruct peptide–opioid receptor interactions, the opioid peptides were labeled with TAMRA at the N terminus.

### Electrophysiology

Whole-cell patch-clamp recordings were made with EPC-8/LIH 1600 amplifier/acquisition system; data were collected using PatchMaster software (all from HEKA, Lambrecht/Pfalz, Germany). Current was recorded at a holding potential of −100 mV, unless otherwise indicated. Current traces were sampled at 10 kHz and filtered online at 3 kHz. Patch electrodes (2–3 MΩ) were filled with a solution containing (in mM): 120 KF and 20 Tris-Cl (adjusted to pH 7.3 with KOH). Extracellular solution contained (in mM): 130 NaCl, 5 KCl, 2 MgCl_2_, 2 CaCl_2_ and 20 HEPES/NaOH (pH 7.4). In experiments where the NMDG-HEPES solutions were used, the pipette solution contained (in mM): 130 NMDG and 10 HEPES (adjusted to pH 7.3 with HF). Extracellular NMDG-HEPES solution contained (in mM): 90 NMDG, 180 HEPES and 2 CaCl_2_ (pH 7.4).

Fully automated ‘jumping table' setup (PharmaRobot, Kiev, Ukraine) was used for applications of external solutions (see Lishko *et al.*^65^). Continuous flow of solution with velocity 0.5 *μ*l/(*s* × mm^2^) was applied to the cell over the entire time of recording.

### DFA of cellular membrane noise

DFA is a method for determination of statistical self-affinity of a signal. It was first introduced by Peng *et al.*^44^ to analyze signals that are characterized by non-stationary statistics. Since then, DFA is widely used in many areas including physiology.^66^ Application of the DFA method for the analysis of holding current fluctuations is briefly formulated as follows. First, the current values sampled at 10 kHz (of total length *N*) are integrated, 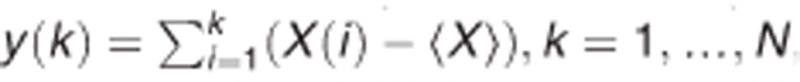, where *X*(*i*) is the i-th holding current sample and *〈X〉* is the average of holding current. Next, the integrated time series is divided into *t* = int(*N*/*L*) nonoverlapping windows of length, *L*. In each window, empirically found tenth-order polynomial (denoted by *y*_*L*_(*k*)) representing the trend in that window is best fitted to the data, and the root-mean-square fluctuation of this integrated and detrended signal is calculated as: 
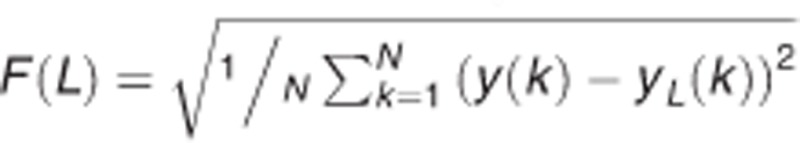
. This computation is repeated over all window scales to provide a relationship between *F*(*L*), the average fluctuation as a function of window size *L*, which reflects the time of observation. The system with power-law correlation obeys *F*(*L*)∝*L*^*α*^, where *α* is the slope of the line relating log(*F*(*L*)) and log(*L*), and is termed the fluctuation or scaling exponent. All calculations described above were made using the DFA algorithm coded by Joe Mietus, C-K Peng and George B Moody, and are available online (http://www.physionet.org/physiotools/dfa/dfa.c) under a GNU General Public License.

### Statistical analysis

All values were expressed as mean±S.D. Differences between means were analyzed using one-way analysis of variance with Bonferroni *post hoc* test. All statistical analyses were performed using the Origin software (OriginLab Corporation, Northampton, MA, USA) with significance set at *P*<0.05.

## Figures and Tables

**Table 1:** Amino-acid sequences and properties of dynorphins

**Peptide**	**Sequence**	**Charge at pH 7.0**	**Basic residues^a^ (%)**	**Hydrophobic residues^b^ (%)**
Dyn A	YGGFLRRIRPKLKWDNQ	+4	30	41
Dyn B	YGGFLRRQFKVVT	+3	23	31
Big Dyn	YGGFLRRIRPKLKWDNQKRYGGFLRRQFKVVT	+9	31	34

a R, K and H.

b V, I, L, M, F, W, Y, P, A

## Abbreviations

AMP: anti-microbial peptide
ASIC: acid-sensing ion channel
Big Dyn: big dynorphin
CLSM: confocal laser scanning microscopy
CPP: cell-penetrating peptide
DFA: detrended fluctuation analysis
DMEM: Dulbecco's modified Eagle's medium
DRG: dorsal root ganglia
Dyn A: dynorphin A
Dyn B: dynorphin B
FBS: fetal bovine serum
FCS: fluorescence correlation spectroscopy
HEPES: 4-(2-hydroxyethyl) piperazine-1-ethanesulfonic acid
MEM: minimum essential medium Eagle's solution
NMDG: *N*-methyl-d-glucamine
PCH: photon counting histogram
PDYN: prodynorphin
RNA: ribonucleic acid
TAMRA: carboxytetramethylrhodamine
TRITC: tetramethylrhodamine
