# Supplementary material for: Plasma membrane poration by opioid neuropeptides: a possible mechanism of pathological signal transduction
Source: Cell Death Dis. 2015 Mar 12;6(3):e1683–. doi: 10.1038/cddis.2015.39 (PMC4385918; doi:10.1038/cddis.2015.39)

# **Online [supplemental](http://www.plosbiology.org/article/info%3Adoi%2F10.1371%2Fjournal.pbio.1001604#s5) material**

# Materials and Methods

**Peptides.** Big Dyn, Dyn A and Dyn B synthesized by Innovagen AB, Sweden*,* were purified by reversed-phase chromatography on Vydac C18 218 TP 1022 and Sephasil C8 columns in a 0.1% trifluoroacetic acid acetonitrile/water solvent system and finally by gel filtration on a Superdex column in 1 M acetic acid. They were analyzed by analytical reversed-phase chromatography and matrix-assisted laser desorption ionization-time of flight mass spectrometry (MALDI-TOF MS). The purity of all peptides was 98%.

TAMRA*-*Big Dyn (TAMRA-YT-YGGFLRRIRPKLKWDNQKRYGGFLRRQFKVVT, C210H312N60O45 , M = 4397.20), TAMRA*-*Dyn A (TAMRA-YQ-YGGFLRRIRPKLKWDNQ, C124H175N33O27 , M = 2559.98) and TAMRA*-*Dyn B (TAMRA-YT-YGGFLRRQFKVVT, C99H135N23O21 , M = 1983.32) purchased from Biomatik USA, Wilmington, DE were purified to level > 98% by the producer using preparative reversed-phase high performance liquid chromatography with gradient elution of water/acetonitrile and 0.1% trifluoroacetic acid. The molecular mass of the peptides was determined by Electrospray ionization-mass spectrometry. Fresh peptide solutions were prepared before each experiment from the powdered substance stored at -20°C, added as 5-15 µL aliquots to the culture medium (300 µL) which then was gently mixed.

# Background on FCS

Fluorescence Correlation Spectroscopy (FCS) is a quantitative method with single-molecule sensitivity that can provide information about the concentration, transporting coefficients and the size of fluorescent molecules; kinetic rate constants and equilibrium association/dissociation constants for interactions of fluorescent molecules and rheological properties of the medium, such as the viscosity and/or flow, in which the fluorescent molecules reside [^1^](#_ENREF_1).

To perform FCS measurements, laser light is sharply focused in the sample through a microscope objective (***Supplementary Figure S5 a***). In this way, a diffraction-limited volume element is formed in the sample and fluorescent molecules residing in the illuminated volume element will be excited. The volume from which fluorescence is detected is reduced by a pinhole (confocal aperture) in the image plane.

Thereby out-of-focus light is rejected, enabling signal collection from an observation volume element that can be approximated by a prolate ellipsoid that is, depending on the wavelength of incident light, about 400-650 nm wide and 1-2 µm (***Supplementary Figure S5 a***, insert). This is crucial for reducing the background signal, which is a prerequisite for quantitative analysis with single-molecule sensitivity. The emitted fluorescence is spectrally selected and transmitted to the detector, which responds with an electrical pulse to each detected photon. The number of pulses originating from the detected photons that is recorded during a specific time interval (binning), corresponds to the measured light intensity. In an FCS experiment, fluctuations in fluorescence intensity in time are registered (***Supplementary Figure S5 b***). Using autocorrelation analysis, the fluorescence intensity fluctuations are examined yielding autocorrelation curves if the fluctuations are not random (***Supplementary Figure S5 c***). The experimentally derived autocorrelation curves are fitted in order to derive the molecular numbers and characteristic times for processes contributing to the fluorescence intensity fluctuations [^1^](#_ENREF_1).

FCS measurements were performed using the same optical pathway as for imaging (described above). Fluorescence intensity fluctuations were recorded in a series of 10 consecutive measurements, each measurement lasting 10 s. Temporal autocorrelation analysis was used to analyze the fluorescence intensity fluctuations in order to determine the concentration and the diffusion time of the investigated species in water, cell culturing medium, plasma membrane, cell nucleus and the cytoplasm.

Aqueous solution of TAMRA was used as a reference (***Supplementary Figure S6*)**. Autocorrelation curves recorded for TAMRA in water were fitted using a single-component model for free three-dimensional (3D) diffusion and triplet formation:

**(1)**

In equation (1), *N* is the average number of molecules in the observation volume element; τ*_D_*_,TAMRA_ is the diffusion time of TAMRA, *w_xy_* and *w_z_* are the distances from the center of the laser beam focus in the radial and axial directions, respectively, at which the collected fluorescence intensity has dropped by a factor of e^2^ compared to the maximum intensity; *T* is the average equilibrium fraction of molecules in the triplet state and τ*_T_* the triplet correlation time, related to the rate constants for intersystem crossing and triplet decay. The diffusion time of TAMRA in aqueous solution was determined to be τ*_D_*_,TAMRA_ = (45 ± 5) µs, and the structural parameter was determined to be 7. In all measurements the triplet state occupancy was < 15 %.

In order to assess the amount of free TAMRA, autocorrelation curves recorded in the aqueous solution of TAMRA-dynorphin peptides were fitted using an autocorrelation function derived for a model describing free 3D diffusion of two components and one triplet state:

|  | **(2)** |
| --- | --- |

In equation (2), *N* is the average number of fluorescent molecules, TAMRA and TAMRA-dynorphins, in the observation volume element; *y* is the fraction of free TAMRA molecules; τ*_D_*_TAMRA_ is the diffusion time of free TAMRA and τ*_D_* is the diffusion time of TAMRA-dynorphin. In the fitting analysis, the diffusion time of TAMRA was fixed to the values determined using TAMRA alone, τ*_D_*_,TAMRA_ = 45 µs, and the structural parameter equal to 7. In this way, the contribution of free TAMRA was determined to be < 5 %.

Fluorescence intensity fluctuations recorded at the plasma membrane have contribution from intersystem crossing; free diffusion in the medium, just above the plasma membrane and two-dimensional diffusion in the plasma membrane. Consequently, the following 2-component:

|  | **(3a)** |
| --- | --- |

or 3-component:

|  | **(3b)** |
| --- | --- |

model functions were used for fitting the experimentally derived autocorrelation curves. The 3-component model **(3b)** was used for fitting only when the fitting with a 2-component model **(3a)** was not satisfactory.

Experimental autocorrelation curves (**Figure 1D, b.** and **d.**) fitted using equation (2) for measurements in the medium and (3a) or (3b) for measurements in the plasma membrane, showed that he average number of TAMRA-Dyn A molecules in the medium was N_med_ = (20 ± 2). Two components with different characteristic times were observed in the bulk medium. The contribution of the first component was determined to be *y*_1_ = (1 - *y*_2_) = (0.8 ± 0.1) and the corresponding diffusion time was τ_D1_ = (65 ± 10) µs, as compared to *y*_2_ = (0.2 ± 0.1) and τ_D2_ = (450 ± 150) µs for the second component, indicating that a fraction of TAMRA-Dyn A molecules has reacted with protein molecules/debris in the cell culture medium. In comparison, the average number of TAMRA-Dyn A molecules at the plasma membrane was determined to be N_pm_ = (105 ± 15). As discussed around equation (**3b**), three processes contributed to the fluorescence intensity fluctuations: the singlet-triplet state transition, characterized by T = (0.08 ± 0.05) and τ_T_ = (4 ± 3) µs; TAMRA-Dyn A movement in the medium in the imminent vicinity of the plasma membrane: *y*_1_ = (1 – *y*_2_ – *y*_3_) = (0.3 ± 0.1) and τ_D1_ = (65 ± 20) µs; and TAMRA-Dyn A movement in the plasma membrane having contribution from two processes with markedly different characteristic times: *y*_2_ = (0.32 ± 0.08) and τ_D2_ = (2.2 ± 0.5) ms, and *y*_3_ = (0.38 ± 0.12) and τ_D3_ = (0.13 ± 0.05) s. The process characterized by τ_D2_ = (2.2 ± 0.5) ms, yielding an apparent diffusion coefficient D_2_ that is of the order of 10^-12^ m^2^ s^-1^ may reflect free 2D diffusion of TAMRA-Dyn A in the plasma membrane. The slowest process characterized by the longest diffusion time τ_D3_ = (0.13 ± 0.05) s, yielding an apparent diffusion coefficient D_3_ that is of the order 10^-14^ m^2^ s^-1^, may represent the dynamics of larger TAMRA-Dyn A assemblies, moving very slowly in the plasma membrane. Similarly, autocorrelation analysis of TAMRA-Big Dyn data showed that the average number of TAMRA-Big Dyn molecules at the plasma membrane N_pm_ = (119 ± 19) was higher than in the medium, N_med_ = (45 ± 6). Similarly to TAMRA-Dyn A, three processes contributed to the fluorescence intensity fluctuations: the singlet-triplet state transition, characterized by T = (0.10 ± 0.05) and τ_T_ = (5 ± 3) µs; TAMRA-Big Dyn movement in the medium in the imminent vicinity of the plasma membrane: *y*_1_ = (1 – *y*_2_ – *y*_3_) = (0.5 ± 0.1) and τ_D1_ = (80 ± 20) µs; and TAMRA-Big Dyn movement in the plasma membrane having contribution from two processes with markedly different characteristic times: *y*_2_ = (0.39 ± 0.08) % and τ_D2_ = (1.8 ± 0.4) ms, and *y*_3_ = (0.11 ± 0.05) and τ_D3_ = (0.43 ± 0.15) s. Analogously to TAMRA-Dyn A, these diffusion times may reflect free 2D diffusion of TAMRA-Dyn A (τ_D2_) and larger TAMRA-Dyn A assemblies, possibly pores.

# Supplementary references

# 1. Vukojevic V, Pramanik A, Yakovleva T, Rigler R, Terenius L, Bakalkin G. Study of molecular events in cells by fluorescence correlation spectroscopy. *Cellular and molecular life sciences : CMLS* 2005 Mar; 62(5): 535-550.

# 2. Elson EL. Brief introduction to fluorescence correlation spectroscopy. *Methods in enzymology* 2013; 518: 11-41.

# 3. Bacia K, Schwille P. Fluorescence correlation spectroscopy. *Methods in molecular biology* 2007; 398: 73-84.

# 4. Hugonin L, Barth A, Graslund A, Peralvarez-Marin A. Secondary structure transitions and aggregation induced in dynorphin neuropeptides by the detergent sodium dodecyl sulfate. *Biochimica et biophysica acta* 2008 Nov; 1778(11): 2580-2587.

# Supplementary figure legends

**Figure S1.** Autocorrelation curves recorded in the bulk cell culture medium after 30 min incubation. FCS analysis showed that the concentration of dynorphin peptides in the bulk medium is different, as evident from differences in the amplitudes of the corresponding autocorrelation curves. Initial concentrations of all three peptides were 100 nM, but after 30 min incubation TAMRA-Big Dyn (magenta) and TAMRA-Dyn A (green) concentrations in the medium decreased from the initial 100 nM to 50 and 70 nM, respectively, while the amplitude of TAMRA-Dyn B (blue) showed that the concentration in the bulk medium is 100 nM.

**Figure S2**. Application of Big Dyn did not induce the appearance of discrete current levels. (**A**) Application of 100 nM of Big Dyn at 240 s induced characteristic cell membrane noise slowly increasing in time until Big Dyn removal at 1680 s. (**B**) All points histograms sampled every 30 s in course of time are shown. (**C**) Histograms calculated for control (120 s) and under 100 nM of Big Dyn (at 1200 s and 1500 s).

**Figure S3**. Spectral analysis of the Big Dyn-induced effect. (**A**) The same experiment as in **Figure S2A**. (**B**) Corresponding spectrogram of the data shows growing 1/f-like noise in response to Big Dyn application. (**C**) Log-log plots of power spectral density at control (120 s) and under 100 nM of Big Dyn (at 1200 s and 1600 s).

**Figure S4**. Non-opioid nature of dynorphin-induced current fluctuations. (**A**) 1 µM Big Dyn induced holding current surges under 100 µM of naloxone as well as in control conditions. (**B**) DynA 2-17 induces holding membrane noise quantitatively the same with the effect of *wt* DynA.

**Figure S5**. (**A**) Schematic presentation of the FCS setup. (**B**) Schematic drawing of a PC12 cells indicating locations at which FCS measurements were performed (not drawn to scale). (**C**) Upper panel, fluorescence intensity fluctuations recorded at the plasma membrane of a PC12 cell. Lower panel, temporal autocorrelation analysis of the fluorescence intensity fluctuations yields an autocorrelation curve (black line), which is thereafter fitted with a corresponding autocorrelation function (red line) to determine molecular numbers and characteristic decay times for the underlying processes.

**Figure S6**. (**A**) Autocorrelation curves of TAMRA-Big Dyn, TAMRA-Dyn A, TAMRA-Dyn B and TAMRA in aqueous solution. (**B**) Autocorrelation curves normalized to the same amplitude, G_n_(τ) = 1 at τ = 1 × 10^-5^ s. The diffusion times were determined by fitting, using equation (1) for TAMRA and equation (2) for the TAMRA-labeled dynorphin peptides: τ*_D_*_,TAMRA_ = (45 ± 5) µs, τ*_D_*_,TAMRA-Dyn A_ = (65 ± 5) µs, τ*_D_*_,TAMRA-Dyn B_ = (70 ± 5) µs. TAMRA-Big Dyn in aqueous solution is prone to aggregation, showing approximately equal amounts of monomeric and aggregated peptides, *y* = 1 - y = 0.5, characterized with diffusion times, τ*_D_*_,TAMRA-Big Dyn, mono_ = (80 ± 5) µs and τ*_D_*_,TAMRA-Big Dyn, aggr_ = (1.0 ± 0.1) ms, respectively. The diffusion time of Dyn B is somewhat longer than the diffusion time of Dyn A, which may arise because of differences in their secondary structure. Indeed, while Dyn A shows propensity for α-helix formation, Dyn B does not, but rather forms β-sheets [^4^](#_ENREF_4).

**Figure S1**


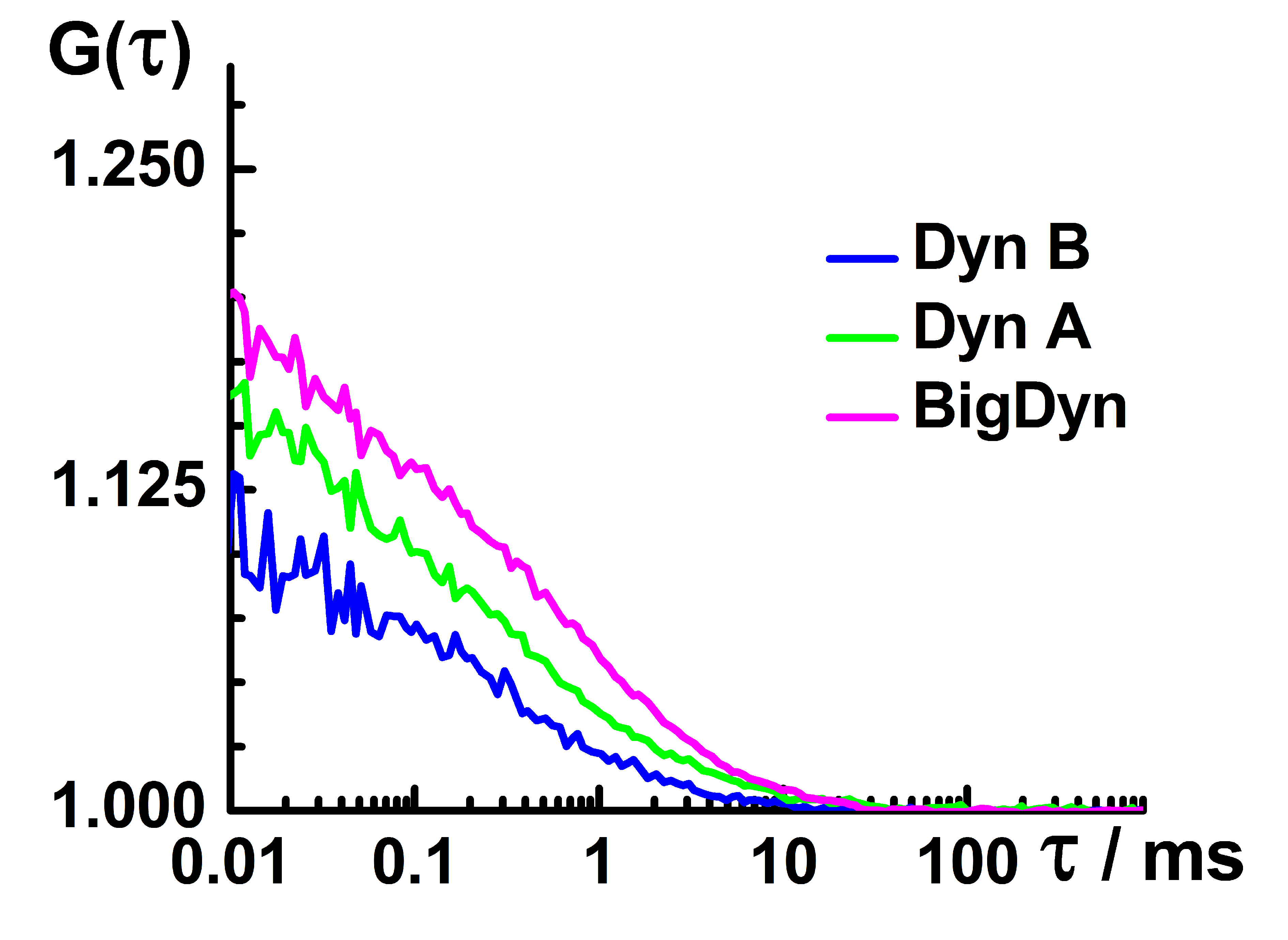


**Figure S2**


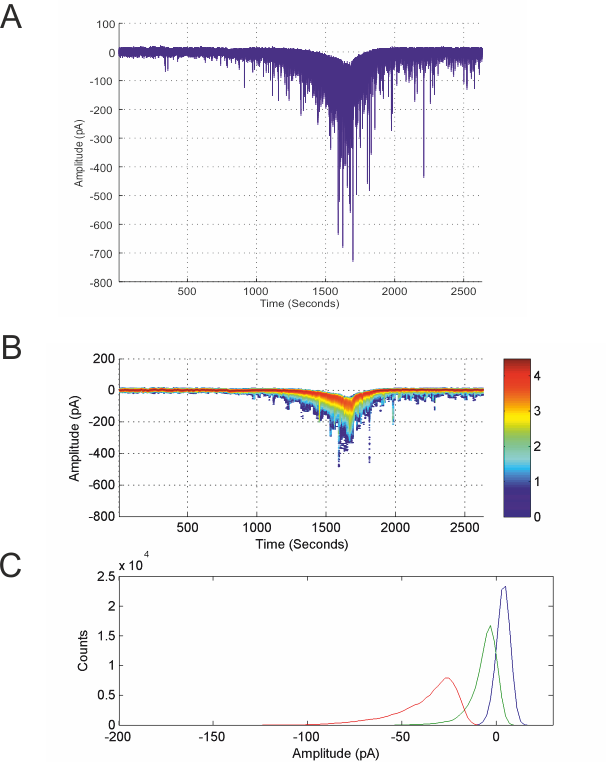


**Figure S3**


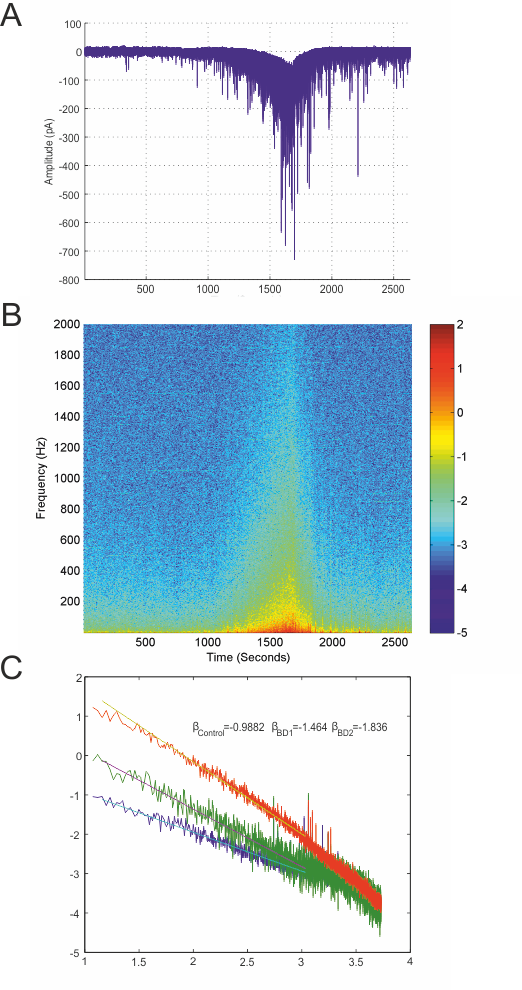


**Figure S4**


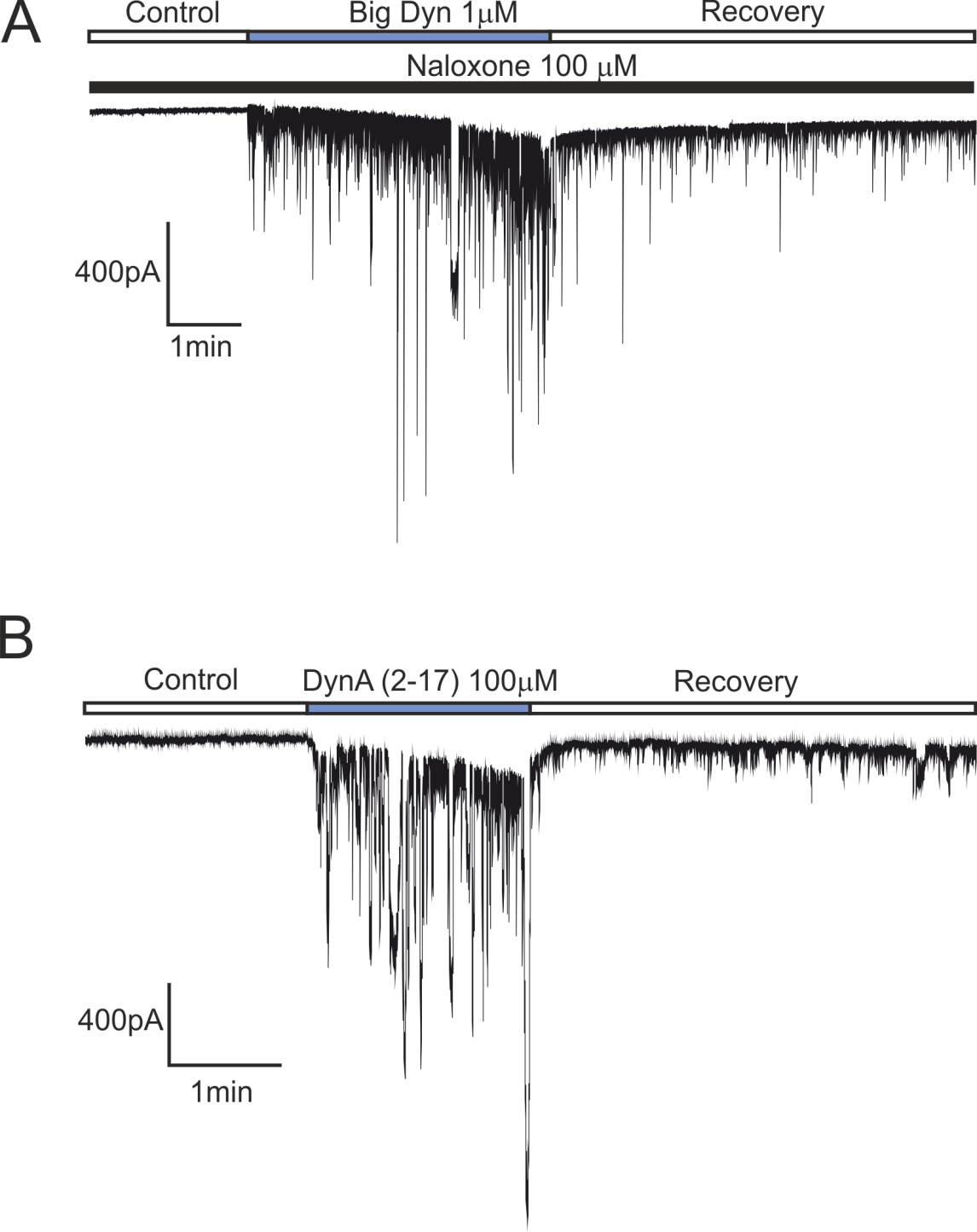


**Figure S5**


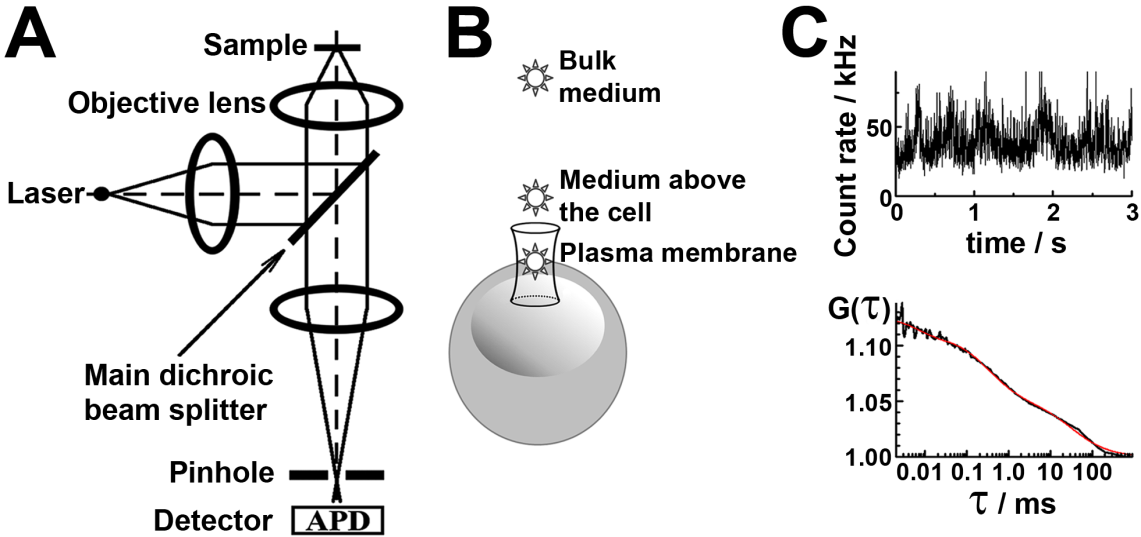


**Figure S6**


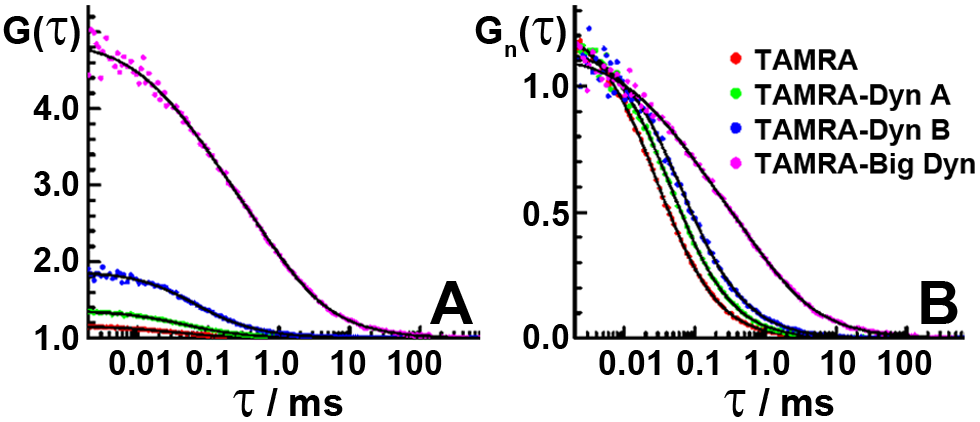

Supplement: Supplementary Information [file cddis201539x1.doc]
